# Complete Genome Sequences of the Potential Zoonotic Pathogens Staphylococcus felis and Staphylococcus kloosii

**DOI:** 10.1128/genomeA.00404-18

**Published:** 2018-05-17

**Authors:** Anne Caroline Mascarenhas dos Santos, Ruili Jie, Helena Antunes Godoy, Manuela Alves, Jean-François Pombert

**Affiliations:** a Department of Biology, Illinois Institute of Technology, Chicago, Illinois, USA

## Abstract

Coagulase-negative staphylococci (CoNS) are opportunistic pathogens frequently encountered in nosocomial infections. Animal-associated CoNS pose a zoonotic risk and constitute a potential reservoir for virulence and antimicrobial resistance genes. To improve our knowledge of animal-associated CoNS, we sequenced the complete genomes of Staphylococcus felis (ATCC 49168) and Staphylococcus kloosii (ATCC 43959).

## GENOME ANNOUNCEMENT

The genus Staphylococcus is well known for the pathogenic species that it harbors, notably the coagulase-positive species S. aureus (1), but coagulase-negative staphylococci (CoNS) have been increasingly linked to animal diseases (2, 3) and are one of the major causes of hospital-acquired infections (4, 5), such as endocarditis (6) and meningitis (7). CoNS are often categorized as human- or animal-associated staphylococci depending on their regular hosts, but animal-associated staphylococci have been identified as causal agents of human diseases (4, 8). However, the zoonotic risks that they pose remain unclear. To better assess the potential for zoonosis and virulence of these CoNS, we sequenced the complete genomes of S. felis and S. kloosii belonging to the hyicus-intermedius and saprophyticus groups of staphylococcal species (4) and isolated from cats and squirrels, respectively.

The S. felis and S. kloosii strains were obtained from the American Type Culture Collection (ATCC). S. felis (ATCC 49168) and S. kloosii (ATCC 43959) were cultured at 37˚C for 24 h in TSB and NB media, respectively. Bacterial cells were pelleted by centrifugation at 5,000 × *g* for 2 min, and total DNA was extracted from pelleted cells with the MasterPure Gram-positive DNA purification kit (Epicentre, Madison, WI, USA). Oxford Nanopore and Illumina libraries were prepared with PCR barcoding (EXP-LWI001)/2D sequencing (FLO-MIN104) kits and NexteraXT kits, respectively, and sequenced using R9.1 flow cells and high-output cartridges (FC-420-1003) on the MinION (Oxford Nanopore Technologies, Oxford, UK) and MiniSeq (Illumina, San Diego, CA, USA) platforms, respectively. Nanopore and Illumina reads were base called with the Metrichor (August 2016) and Real-Time Analysis version 2.8.6 pipelines, respectively.

Genomes were assembled with SPAdes version 3.7.1 using a hybrid approach combining Illumina and Nanopore reads (9, 10). Genomes and plasmids were circularized by identifying overlapping ends with BLASTn nucleotide homology searches (11) and cutting outside gene loci identified with Prokka version 1.11 (12). Homopolymer errors were corrected by mapping the Illumina reads against the consensus sequences followed by manual curation. Reads were mapped with the addSolexaReads.pl script from the Consed version 29.0 package (13), modified with the minimum score/mismatch penalty set to 50/-9, and the “-gap1_only” option removed to allow mapping to large incorrect homopolymer stretches. Questionable consensus bases were highlighted with Consed’s eponym function and curated manually. Base-corrected consensus sequences of the S. felis (2,479,423 bp, 35.21% G+C, 224.88× coverage) and S. kloosii (chromosome, 2,630,191 bp, 33.08% G+C, 266.82× coverage; plasmid, 8,847 bp, 29.18% G+C, 1,270.84× coverage) genomes were annotated with the NCBI Prokaryotic Annotation Pipeline (14).

### Accession number(s).

The S. felis (ATCC 49168) and S. kloosii (ATCC 43959) complete genome sequences were deposited in GenBank under the accession numbers CP027770 and CP027846/ CP027847 (chromosome/plasmid), respectively.
